# First Successful Delivery after Uterus Transplantation in MHC-Defined Cynomolgus Macaques

**DOI:** 10.3390/jcm9113694

**Published:** 2020-11-17

**Authors:** Iori Kisu, Yojiro Kato, Yohei Masugi, Hirohito Ishigaki, Yohei Yamada, Kentaro Matsubara, Hideaki Obara, Katsura Emoto, Yusuke Matoba, Masataka Adachi, Kouji Banno, Yoko Saiki, Takako Sasamura, Iori Itagaki, Ikuo Kawamoto, Chizuru Iwatani, Takahiro Nakagawa, Mitsuru Murase, Hideaki Tsuchiya, Hiroyuki Urano, Masatsugu Ema, Kazumasa Ogasawara, Daisuke Aoki, Kenshi Nakagawa, Takashi Shiina

**Affiliations:** 1 Department of Obstetrics and Gynecology, Keio University School of Medicine, Tokyo 1608582, Japan; y.matoba0212@gmail.com (Y.M.); madachi1014@keio.jp (M.A.); kbanno@keio.jp (K.B.); aoki@z7.keio.jp (D.A.); 2 Department of Surgery, Division of Gastroenterological and General Surgery, School of Medicine, Showa University, Tokyo 1428555, Japan; yojirony@gmail.com; 3 Department of Pathology, Keio University School of Medicine, Tokyo 1608582, Japan; masugi@z6.keio.jp (Y.M.); emoto@a7.keio.jp (K.E.); 4 Department of Pathology, Shiga University of Medical Science, Shiga 5202192, Japan; ihiro@belle.shiga-med.ac.jp (H.I.); sasamura@belle.shiga-med.ac.jp (T.S.); kogasawara@me.com (K.O.); 5 Department of Pediatric Surgery, Keio University School of Medicine, Tokyo 1608582, Japan; yohei@z7.keio.jp; 6 Department of Surgery, Keio University School of Medicine, Tokyo 1608582, Japan; kmatsubaravs@gmail.com (K.M.); obara.z3@keio.jp (H.O.); 7 Department of Anesthesiology, Saiseikai Kanagawaken Hospital, Kanagawa 2210821, Japan; y_saiki_z7@yahoo.co.jp; 8 Research Center for Animal Life Science, Shiga University of Medical Science, Shiga 5202192, Japan; itagaki@belle.shiga-med.ac.jp (I.I.); ikuok@belle.shiga-med.ac.jp (I.K.); chiduru@belle.shiga-med.ac.jp (C.I.); nakagawa@belle.shiga-med.ac.jp (T.N.); murase@belle.shiga-med.ac.jp (M.M.); htsuchiya@belle.shiga-med.ac.jp (H.T.); mema@belle.shiga-med.ac.jp (M.E.); 9 Safety Research Center, Ina Research Inc., Nagano 3994501, Japan; hurano1115@gmail.com (H.U.); n_kenshi@ina-research.co.jp (K.N.); 10 Department of Molecular Life Science, Division of Basic Medical Science and Molecular Medicine, Tokai University School of Medicine, Kanagawa 2591193, Japan; tshiina@is.icc.u-tokai.ac.jp

**Keywords:** allogeneic uterus transplantation, cynomolgus macaque, donor specific antibody, humoral rejection, post-transplant lymphoproliferative disorder, uterine transplantation, uterine factor infertility, uterus transplantation

## Abstract

Delivery following uterus transplantation (UTx)—an approach for treating uterine factor infertility—has not been reported in nonhuman primate models. Here, six female major histocompatibility complex (MHC)-defined cynomolgus macaques that underwent allogeneic UTx were evaluated. Antithymocyte globulin and rituximab were administered to induce immunosuppression and a triple maintenance regimen was used. Menstruation resumed in all animals with long-term survival, except one, which was euthanized due to infusion associated adverse reaction to antithymocyte globulin. Donor-specific antibodies (DSA) were detected in cases 2, 4, and 5, while humoral rejection occurred in cases 4 and 5. Post-transplant lymphoproliferative disorder (PTLD) developed in cases 2 and 3. Pregnancy was attempted in cases 1, 2, and 3 but was achieved only in case 2, which had haploidentical donor and recipient MHCs. Pregnancy was achieved in case 2 after recovery from graft rejection coincident with DSA and PTLD. A cesarean section was performed at full-term. This is the first report of a successful livebirth following allogeneic UTx in nonhuman primates, although the delivery was achieved via UTx between a pair carrying haploidentical MHCs. Experimental data from nonhuman primates may provide important scientific knowledge needed to resolve unsolved clinical issues in UTx.

## 1. Introduction

Uterus transplantation (UTx) offers an option for treatment of women with uterine factor infertility to become pregnant. The first delivery of a human infant following UTx was accomplished in 2014 [1]. Although this achievement attracted global attention to research on UTx, it is still at the experimental stages and many clinical and technical issues remain unsolved [2]. Therefore, further animal studies are required to provide important information for establishment of UTx as a clinical application.

Studies on UTx have been performed in mice, rats, rabbits, dogs, pigs, sheep, and nonhuman primates [3]. Despite the reports of nonhuman primates since 2010 [2], published reports are rare [4,5,6,7,8,9,10,11,12,13,14]. Furthermore, despite deliveries in rats [15] and sheep [16], delivery following allogeneic UTx in nonhuman primates is yet to be attained.

Nonhuman primate models, including macaques, are frequently used for clinical and nonclinical research due to the phylogenetic proximity and anatomic and physiologic similarities of their reproductive organs to humans. The phylogenetic proximity of the immune and reproductive systems allows for the extrapolation of macaque-based organ transplantation and reproductive medicine studies to humans [17]. However, as the size of a female macaque is approximately 3–4 kg—quite small compared to humans—fine surgical techniques are required. Thus, validation of medical and technical issues associated with UTx in macaques, with particular reference to immune and reproductive aspects, may provide important preclinical data for resolving issues pertaining to UTx in humans.

Following the first report of a successful delivery following autologous UTx in primates [18], we proceeded to establish cynomolgus macaque allogeneic UTx models. As described previously, although we succeeded in surgically ensuring the long-term survival of allogeneic UTx models [14], we were unable to overcome uterine graft rejection following UTx, leading to graft failure likely due to high antigenicity. Therefore, the previous immunosuppressant protocol was modified and enhanced to establish a stable uterine allograft model in cynomolgus macaques.

Moreover, we focused on defining the major histocompatibility complex (MHC) to reduce the antigenicity of the transplanted uterus in cynomolgus macaques. MHC, also known as human leukocyte antigen (HLA) in human, is recognized by lymphocytes and other immune cells, resulting in an immune response following allogeneic transplantation. HLA matching improves graft survival rates following transplantation of organs, such as kidneys and bone marrow, by reducing immunological graft rejection [19,20,21]. Generally, grafts from MHC homozygous donors are immunologically acceptable in MHC-matched heterozygous recipients, whereas grafts containing mismatched-MHCs are recognized and immunologically rejected by the immune cells of the recipient [22]. We have established an MHC genotyping technique based on next generation sequencing of MHC class I and II cDNA in cynomolgus macaques [23,24]. Here, MHC-defined haploidentical recipient and donor pairs selected by this technique exhibited lower graft rejection rate following UTx transplantation during experiments.

The current study presents the outcomes of allogeneic UTx performed on MHC-defined cynomolgus macaques using a modified immunosuppressant protocol and reports the first successful delivery following recovery from graft rejection coincident with donor-specific antibody (DSA) and post-transplant lymphoproliferative disorder (PTLD).

## 2. Materials and Methods

### 2.1. Animals

Twelve female cynomolgus macaques (*Macaca fascicularis*), aged 5–13 years and weighing between 2.67 and 4.07 kg, were used in this study (Table 1). Six recipient and donor pairs with mismatched and haploidentical MHCs and compatible ABO blood types were selected. Negative blood type cross matches and DSAs between donor and recipient were confirmed in advance prior to surgery. All animals were IgG-positive for Epstein–Barr virus. The study was performed in accordance with the recommendations of the Guide for the Care and Use of Laboratory Animals of the National Research Council, and approved by the Animal Care and Use Committee of the Research Center for Animal Life Science, Shiga University of Medical Science, Japan (permit number: 2016-4-8 and 2019-3-12).

### 2.2. MHC Typing

Genotyping of the cynomolgus macaque MHC Mafa class I genes (*Mafa-A* and *Mafa-B*) and Mafa class II genes (*Mafa-DRB*, *Mafa-DQB1* and *Mafa-DPB1*) were performed using previously reported MHC locus-specific primer sets and PCR conditions [23,24]. Total RNA was isolated from peripheral blood cells using TRIzol reagent (Thermo Fisher Scientific, Carlsbad, CA) according to the manufacturer’s instructions. Next, cDNA was synthesized via oligo d (T) primer using the ReverTraAce for reverse transcriptase reaction (TOYOBO, Osaka, Japan) after treating isolated RNA with DNase I (Thermo Fisher Scientific). Semiconductor sequencing of PCR products was conducted using the Ion Torrent system and amplicon sequencing protocol (Thermo Fisher Scientific, Palo Alto, CA, USA). MHC genotypes were assigned by comparing the sequences to known MHC allele sequences via the Immuno Polymorphism Database [25].

MHC genes of the six recipient and donor pairs are shown in Table S1. The cases 1, 4, and 5 involved pairs with mismatched MHC between donor and recipient, while cases 2 (Table 2) and 3 had pairs with haploidentical donor and recipient MHCs.

### 2.3. Uterus Transplantation

**Figure 1:**
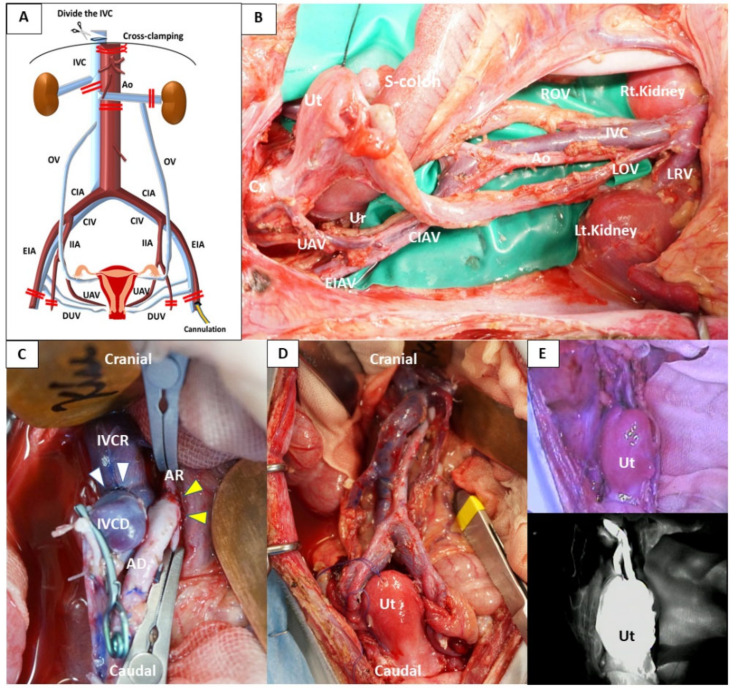
Donor and recipient surgery. (**A**) Schema of abdominal vessels dissected during donor surgery. Cross-clamping was performed by clamping the supraceliac aorta under the diaphragm and dividing the inferior vena cava (IVC) in the pleural space. A perfusion catheter was placed in the unilateral external iliac artery. (**B**) Dissection of the abdominal vessels and tissues surrounding the uterus before procurement of the uterus via donor surgery. Infrarenal aorta/IVC above the left renal vein, iliac and uterine vessels, and ovarian veins were dissected. Subsequently en bloc procurement of the uterus with these vessels and proximal vagina was performed. (**C**) Vascular anastomosis in recipient surgery. End-to-side venous anastomosis between the IVC of the donor and that of the recipient (white triangle) and between the abdominal aorta of the donor and the abdominal aorta of the recipient (yellow triangle). (**D**) Transplanted uterus. Following reperfusion, the color of the transplanted uterus changed from white to red without uterine congestion. (**E**) Intraoperative indocyanine green (ICG) fluorescent angiography in the transplanted uterus. ICG fluorescent angiography showed that there was sufficient blood flow to the transplanted uterus immediately following reperfusion (before (upper panel) and after (lower panel) injection of ICG).

Following sedation with intramuscular (i.m.) ketamine and xylazine, animals were intubated and operated on under general anesthesia with ventilation maintained by isoflurane inhalation (0.5–1.5%; Abbot Japan, Tokyo, Japan). All animals received 25 mg/kg of cefazolin every 3 h as prophylaxis starting at initiation of surgery. In donor surgery, thoraco-laparotomy was performed, and extensive vascular dissection of the abdominal vessels, including infrarenal aorta/inferior vena cava (IVC) above the left renal vein, iliac and uterine vessels, and ovarian veins, was conducted (Figure 1A,B). Superior vesical, pudendal, gluteal, and sacral branches of the iliac vessels were ligated and transected along with all lumbar branches of the infrarenal aorta and IVC, up to the level of the renal vessels. The ovaries of the donors were removed in situ, without the fallopian tubes, to avoid ovarian transplantation from the donor and to maintain the ovarian veins. Prior to organ procurement, a 150 mL of whole blood, diluted via infusion load, was collected from the donor and stored in an anticoagulant bag including a citrate-phosphate-dextrose solution with adenine (Sepacell Integra CA, Kawasumi Laboratories, Inc., Tokyo, Japan). After leukocytes were depleted using leukoreduction filter sets (Sepacell Integra CA, Kawasumi Laboratories, Inc.), blood was irradiated (30 Gy) to prevent graft-versus-host disease. Then, an anticoagulant (heparin, 1000 IU) was administered intravenously 5 min before cross-clamping of the abdominal aorta. The uterine grafts were perfused with 250–300 mL of cold histidine-tryptophan-ketoglutarate solution (HTK^®^; Custodiol^®^, Nordmedica, Gentofte, Denmark) via a perfusion catheter (22-G intravenous needle; Terumo Corp., Tokyo, Japan) into the external iliac artery. Then, the uterus, fallopian tubes, and the proximal vagina were recovered en bloc with the infrarenal aorta/IVC above the left renal vein, iliac and uterine vessels, and ovarian veins. The recipient was subjected in advance to a total simple hysterectomy with preservation of ovaries. Subsequently, the infrarenal abdominal aorta/IVC was exposed at a distance of approximately 2 cm. The uterine graft was brought into the operative field and the proximal vagina of the graft was anastomosed with the vaginal vault of the recipient to fix the uterus in the pelvis. Before vascular anastomoses, heparin (200 IU) was intravenously administered. Recipient surgery consisted of end-to-side anastomosis with the recipient’s and donor’s infrarenal aorta/IVC, performed using continuous sutures (7–0 or 8–0 Prolene^®^, Johnson & Johnson) (Figure 1C). Next, the vascular clamps were released and the graft was reperfused. Following reperfusion, intraoperative indocyanine green (ICG) (Diagnogreen 0.5%; Daiichi Pharmaceutical, Tokyo, Japan) was intravenously injected to confirm the blood supply in the transplanted uterus and blood flow at the site of vascular anastomosis, as previously described [26] (Figure 1D,E). Stored donor blood was transfused into the recipient during vascular anastomosis and/or following reperfusion of the uterine graft, after confirming that the white blood cell count was zero. The retroperitoneum and round ligaments were sutured before closing the abdominal incision. Antibiotics, a proton-pump inhibitor, and aspirin were administered following surgery.

AD, aorta of the donor; Ao, abdominal aorta; AR, aorta of the recipient; CIA, common iliac artery; CIV, common iliac vein; CIAV, common iliac artery and vein; Cx, cervix; DUV, deep uterine vein; EIA, external iliac artery; EIAV, external iliac artery and vein; IIA; internal iliac artery; IVC, inferior vena cava; IVCD, inferior vena cava of the donor; IVCR, inferior vena cava of the recipient; LOV, left ovarian vein; LRV, left renal vein; OV, ovarian vein; ROV, right ovarian vein; UAV, uterine artery and vein; Ur, ureter; Ut, uterus.

### 2.4. Immunosuppressive Management

Severe rejection and antibody mediated rejection induced with DSA production were observed in the previous study [14]. Therefore, rituximab (anti-CD20 monoclonal antibodies) was added to the immunosuppressive protocol (Table 3). Induction treatment of animals consisted of rituximab (2 mg/kg; Rituxan; Genentech, San Francisco, CA, USA) administered approximately 3 weeks prior to surgery (17 to 23 days before surgery) and on postoperative day (POD) 0 (the day of surgery) and antithymocyte globulin (ATG) (20 mg/kg; Thymoglobulin, Genzyme, Cambridge, MA, USA) intravenously administered on POD 0 and POD 2. Maintenance treatment consisted of tacrolimus (TAC) (Prograf; Astellas Pharma, Tokyo, Japan) administered intramuscularly from day −5 with target trough levels of 15 to 20 mg/mL during the first month and tapered thereafter as required. Another maintenance treatment, mycophenolate mofetil (MMF) (40 mg/kg; Cellcept; Chugai Pharmaceutical, Tokyo, Japan) which was orally administered using an orogastric catheter from day −5, twice daily, and adjusted thereafter to exceed trough levels of at least 1 µg/mL. MMF was replaced with azathioprine (1–2 mg/kg; Azanine, Mitsubishi Tanabe Pharma, Osaka, Japan) before attempting impregnation, to avoid potential teratogenic effects of MMF. Methylprednisolone (10 mg/kg; Solu-Medrol; Pfizer, NY, USA) was intravenously injected on POD 0 and then intramuscularly daily starting POD 1. Methylprednisolone dose was gradually tapered and continuously administered at daily doses of 0.2–0.4 mg/kg, until the end of observation. In the event of rejection, steroid pulse therapy with 10 mg/kg methylprednisolone was administered for 2 d and gradually tapered. In case of refractory graft rejection despite conventional steroid pulse treatment, ATG was added. In addition, rituximab was administered when a flow-cross match was positive for DSA and histological findings predicted antibody mediated rejection.

### 2.5. Flow Cytometry

After removing red blood cells with BD Lysing buffer (BD Bioscience, Franklin Lakes, NJ, USA), the peripheral blood cells were stained with fluorescence-conjugated antibodies. Next, 7-aminoactinomycin D (7-AAD; BD Bioscience) was used for dead cell staining. Isotype-control antibodies were used as negative controls. Samples were acquired and analyzed using a CytoFlex S instrument (Beckman Coulter Inc., Brea, CA, USA). The antibodies used in this study are listed in Table S2.

### 2.6. Mixed Lymphocyte Reaction (MLR)

First, 5 × 10^5^ recipient peripheral blood cells collected from macaques before and after transplantation were cultured in an ELISPOT plate (Mabtech AB, Nacka Strand, Sweden) with 5 × 10^5^ donor splenocytes after lysing red blood cells. Culturing was conducted in triplicate or duplicate wells depending on the number of cells. After 3 d of culturing, the number of interleukin-2 (IL-2) positive spots was counted using an ImmunoSpot analyzer (Cellular Technology, Cleveland, OH, USA). Stimulation indices (SI) were calculated by the following formula: number of spots in the blood cell culture plus donor splenocytes/number of spots in the blood cell culture alone.

### 2.7. Donor-Specific Antibody

Heat-inactivated plasma of the transplanted macaques was diluted 10 times in phosphate-buffered saline (PBS; Nacalai Tesque Inc., Kyoto, Japan) before use. The diluted plasma was added to donor splenocytes as a primary antibody. Fluorescein isothiocyanate (FITC)-conjugated goat polyclonal anti-mouse IgG (Nordic Immunological Laboratories, Copenhagen, Denmark) was used as a secondary antibody to detect donor specific IgG. Phycoerythrin conjugated anti-CD20 antibodies (Clone: 2H7, BioLegend, San Diego, CA, USA), and allophycocyanin conjugated anti-CD3 antibodies (Clone: SP34-2, BD Bioscience, San Diego, CA, USA) were also used to identify the cell type of the DSA attached cells. Cells were analyzed using flow cytometry.

### 2.8. Postoperative Observation

Following surgery, the animals were separately accommodated in single cages and their general conditions were evaluated daily. Laboratory assessments, including hematology, blood chemistry, and tacrolimus levels, were conducted twice a week during the first 2 postoperative weeks and weekly for 2 months thereafter, following which the assessments were tapered as required. To detect potential rejection following surgery, transabdominal ultrasonography and transvaginal biopsies of transplanted uterine tissues were performed under anesthesia using a puncture needle (BARD MAX-CORE; BD [C.R. Bard, Inc.], Tempe, AZ, USA). Flow cytometry, DSA, and MLR analyses were also performed as required. In case of irreversible rejection, weight loss, or untreatable illness, or when deemed to be necessary by veterinary staff and investigators, animals were euthanized.

### 2.9. Histological Evaluation and Rejection Criteria

**Figure 2:**
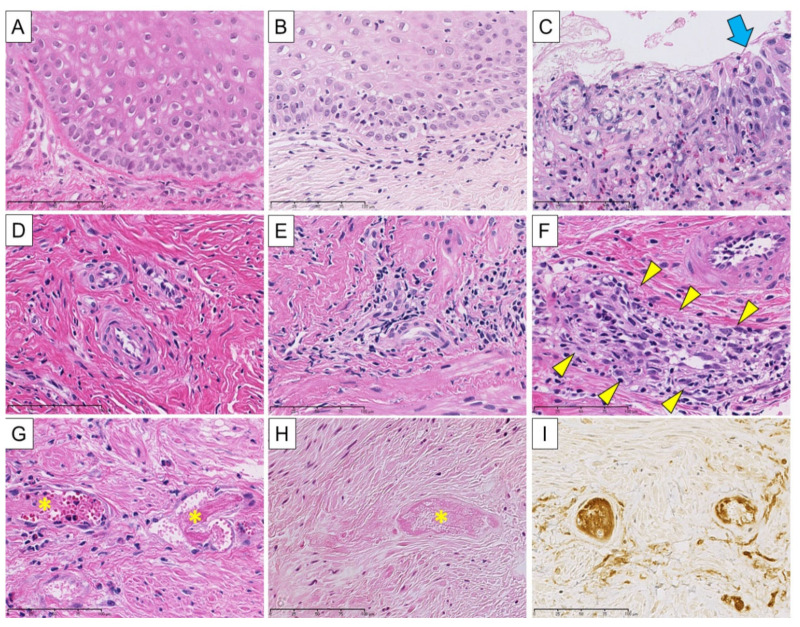
Histologic variables of cellular and humoral rejection in cynomolgus macaques following uterus transplantation. (**A**) Normal squamous epithelium of the cervix <E0>. (**B**) Focal vacuolar alteration of the basal cell layer with mild junctional lymphocytic infiltrates <E1>. (**C**) Desquamative change of squamous epithelium. A small amount of residual epithelium is highlighted by a blue arrow <E3>. (**D**) Blood vessels and fibromuscular stroma with no significant inflammation <S0 and A0>. (**E**) Perivascular inflammation without endotheliitis <S1>. (**F**) Inflammation with endotheliitis in addition to perivascular inflammation, subendothelial lymphocytic infiltration is noted (yellow arrowheads). Venous endothelium demonstrates desquamative change and nuclear swelling <S2 or S3>. (**G**) Fibrin thrombi (*) in the small vessels without tissue necrosis <A1>. (**H**) Fibrin thrombi (*) in the small vessels surrounded by coagulative necrosis <A2>. (**I**) Immunohistochemistry for C4d using the same specimen from (**H**). Positive C4d immunostaining was seen along subendothelial spaces and in venous lumen. (**A**–**H**) H&E stain, bar = 100 µm. (**I**) Immunohistochemistry for C4d, bar = 100 µm.

Uterine biopsy tissues were fixed in 10% neutral buffered formalin, embedded in paraffin, and stained with hematoxylin and eosin. As described previously [14], an automated staining system involving a Bond Polymer Refine Detection kit with an EBER Probe that detects Epstein–Barr virus-encoded mRNA was used for in situ hybridization and for immunostaining CD20 and C4d. Allograft rejection was histopathologically assessed and carefully validated by two pathologists (Y.M. and K.E.) who were blinded to the detailed clinical course (Table 4 and Figure 2). This validation was based on our rejection criteria, which partially referred to the grading system proposed by a Swedish group for monitoring rejection [27].

### 2.10. Intracytoplasmic Sperm Injection and Embryo Transfer

**Figure 3:**
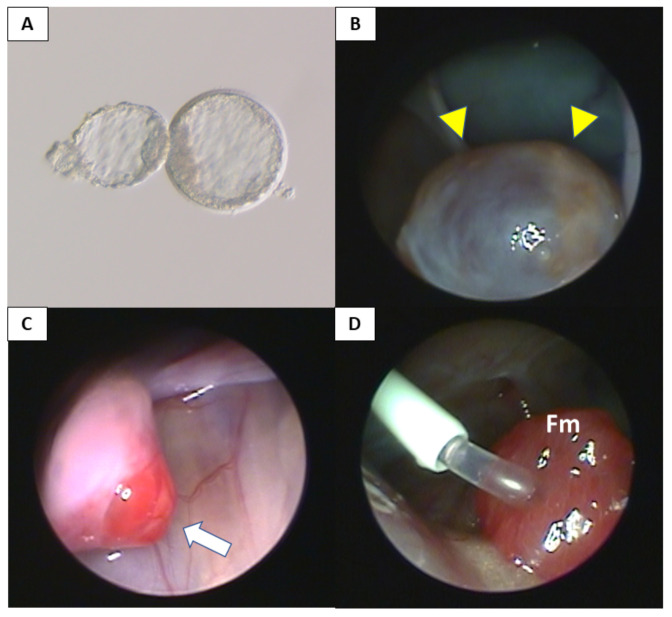
Intracytoplasmic sperm injection and embryo transfer to the grafted uterus. (**A**) Blastocyst-stage embryo for embryo transfer is hatching from zona pellucida. (**B**) Follicular development of ovarian preovulatory follicle (yellow triangles) was observed via laparoscopy. (**C**) Ovulation (white arrow) was confirmed using laparoscopy. (**D**) Embryo intrafallopian transfer was performed after confirmation of ovulation. Embryos were inserted into the fallopian tube through a catheter. Fm, fimbria of oviduct.

After confirming periodic menstruation following surgery, artificial insemination by a third party donor was attempted in order to attain pregnancy, although sperm was inserted via external os of the grafted uterus using a catheter, due to the cervical canal of cynomolgus macaques being bended and tortuose, preventing the insertion of a catheter into the uterine cavity. When pregnancy was not achieved using this procedure at least three times, intracytoplasmic sperm injection (ICSI) and embryo transfer (ET) were conducted (Figure 3). The procedures that were performed on cynomolgus monkeys for oocyte collection, ICSI, in vitro culturing of the embryo and ET were as follows [28,29]: In the indoor artificial breeding of monkeys as laboratory animals, the usage of fertilized oocytes belonging to a third party for assisted reproduction technologies is the most common as well as the most efficient practice [30]. Therefore, oocyte collection was performed from third party female cynomolgus macaques. Third party female cynomolgus macaques (aged 5–7 years) marked for oocyte collection were downregulated by subcutaneous injection of a GnRH antagonist (0.9 mg/animal; Leuplin; Takeda, Osaka, Japan). Ovarian stimulation with follicle-stimulating hormone (Gonapure, Aska Pharmaceutical, Tokyo, Japan) was performed by embedding an implantable and programmable micro-infusion pump (iPRECIO, Primetech Corporation, Tokyo, Japan) subcutaneously. Follicular aspiration with a 3 mm laparoscope attached to a video system was performed 40 h post-hCG (Gonatropin (for animal), Aska Pharmaceutical, Tokyo, Japan,), following which mature oocytes were collected and subjected to ICSI with fresh sperm collected via electric stimulation of the penis of a well-trained male macaque, with no anesthesia. ICSI-embryos were cultured for 7–8 d until transfer or vitrification. Embryo vitrification and thawing were performed according to the method described by Yamasaki et al. [28] and blastocyst-stage embryos were selected. Ovarian preovulatory follicles, or ovulation points, of the recipients were confirmed using laparoscopy and embryo intrafallopian transfer using laparoscopy for recipients was conducted within 0 to 7 d after ovulation. Transabdominal ultrasonography was performed 3 weeks following embryo transfer to detect pregnancy, by confirming a gestational sac and fetal cardiac activity. Anesthesia, during all laparoscopy and ultrasound scan procedures, was provided by intramuscular injection of 5 mg/kg ketamine hydrochloride (Ketalar; Daiichi Sankyo, Co. Ltd., Tokyo, Japan) in conjunction with 1 mg/kg xylazine hydrochloride (Celactal; Bayer HealthCare, Osaka, Japan).

## 3. Results

The outcomes of clinical course in these animals are summarized in Table 5.

### 3.1. Surgical Outcomes of Uterus Transplantation

Surgical parameters of the six uterus transplantations are shown in Table S3. Five recipient animals (cases 1–5) survived for more than 3 months following surgery without any surgical complications; of these, cases 1 and 2 survived for more than 3 years after surgery. Because of an infusion associated reaction in case 6 due to ATG administration during the surgery, the animal was euthanized after closing the abdomen. In regard to organ perfusion, gross findings in all donor animals indicated that the color of all abdominal organs had changed from red to white, confirming favorable perfusion to all organs. Immediately following reperfusion, all recipients displayed good uterine blood flow, without uterine congestion (Figure 1D). ICG fluorescent angiography performed immediately after reperfusion also showed that the blood flow into the whole transplanted uterus and the site of vascular anastomosis was sufficient (Figure 1E).

### 3.2. Changes of Trough Levels of Immunosuppressants

**Figure 4:**
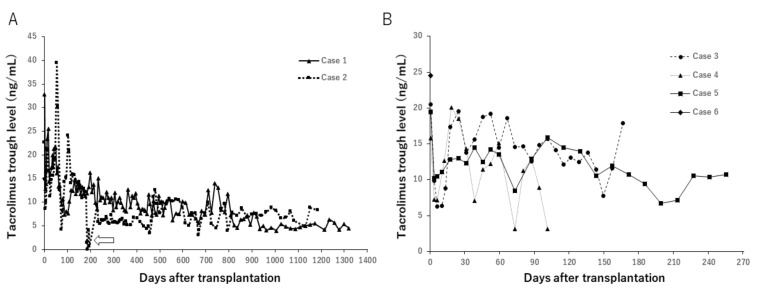
Change of trough levels of tacrolimus in case 1 and 2 (**A**) and case 3 to 6 (**B**). In case 2, TAC was withdrawn from POD 191 to 220 due to PTLD development, resulting in occasional low trough levels during the period (arrow).

Changes in trough levels of tacrolimus (TAC) during maintenance treatment are shown in Figure 4.

### 3.3. Rejection Episodes and Immunosuppressive Treatment

**Figure 5:**
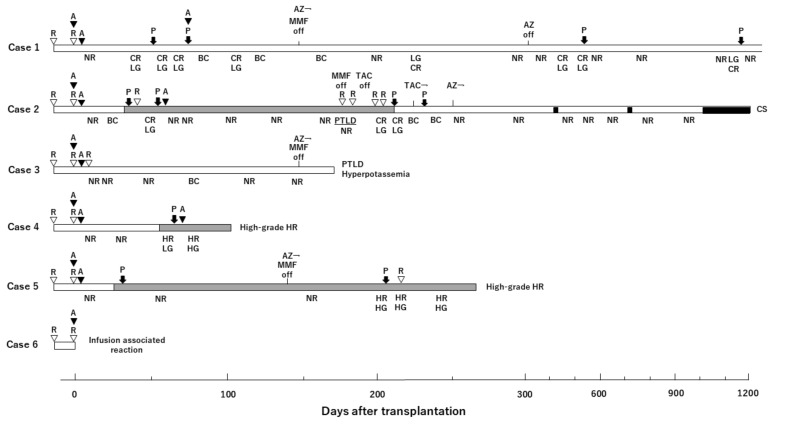
Rejection episodes and immunosuppressive treatment. Results of histopathological examination, immunosuppressive treatment, and cause of sacrifice are shown. Horizontal bars represent the length of animal survival following uterus transplantation. Results of uterine biopsy are depicted under each horizontal bar (NR, no rejection; BC, borderline change; CR, cellular rejection; HR, humoral rejection; LG, low-grade; HG, high-grade). Immunosuppressive treatments are depicted on each horizontal bar (R: rituximab, open triangle; A: antithymocyte globulin, closed triangle; P: steroid pulse treatment, closed arrow; MMF, mycophenolate mofetil; AZ, azathioprine; TAC, tacrolimus) The causes of sacrifice are shown at the end of the survival bar. The presence, including period of serum donor-specific antibody (DSA) (gray fill) and pregnancy (black fill) are indicated within the bars. Case 3 suddenly died due to PTLD and hyperpotassemia although the clinical course seemed to be satisfactory with no rejection and recovery of periodic menstruation. Case 6 was euthanized after closing the abdomen due to infusion associated reaction which occurred after administering antithymocyte globulin during surgery. PTLD, Post-transplant lymphoproliferative disorder; C, caesarian section.

The detailed postoperative course showing rejection and immunosuppressive treatment are summarized in Figure 5.

In case 1, which had mismatched donor and recipient MHCs, a uterine biopsy on postoperative day (POD) 48 showed remarkable perivascular lymphocyte infiltration and endotheliitis, indicating low-grade cellular rejection. Consequently, steroid pulse therapy was used. After a biopsy in POD 74 showed features associated with sustained rejection, additional steroid pulse therapy and ATG were administered. These treatments improved the uterine graft to borderline change in POD 131, with recovery of periodic menstruation. Therefore, MMF was replaced with azathioprine on POD 142. Thereafter, low-grade cellular rejection was detected in POD 487, 503, and 1147, which were treated with steroid pulse therapy. On POD 1200, uterine viability with periodic menstruation was maintained without rejection.

In case 2, which had haploidentical donor and recipient MHCs, a biopsy of POD 32 showed perivascular lymphocyte infiltration with an apoptotic body and negative C4d staining, indicating borderline change. Steroid pulse therapy was administered. Moreover, rituximab (2 mg/kg) was added because DSA was detected. The histopathological findings of a biopsy conducted on POD 48 showed remarkable perivascular inflammation with definite endotheliitis. Therefore, additional steroid pulse therapy and ATG were administered, which led to the disappearance of pathological findings suggesting cellular rejection. Monthly biopsies indicated no histological signs of rejection for 4 months, despite positive DSA persistence and PTLD development on POD 180. For treatment of PTLD, cessation of MMF and TAC was conducted, resulting in the induction of low-grade cellular rejection on POD 212. Complete clearance of rejection with recovery of PTLD was achieved via repetitive steroid pulse therapy and restarting TAC.

In case 3, whose MHC was haploidentical to that of the donor, monthly biopsies indicated that there were no histological signs of uterine rejection or DSA production with the recovery of periodic menstruation until 5 months. However, sudden death occurred on POD 170 induced by PTLD and hyperpotassemia that elevated potassium levels up to 7.7 mEq/L on POD 166.

**Figure 6:**
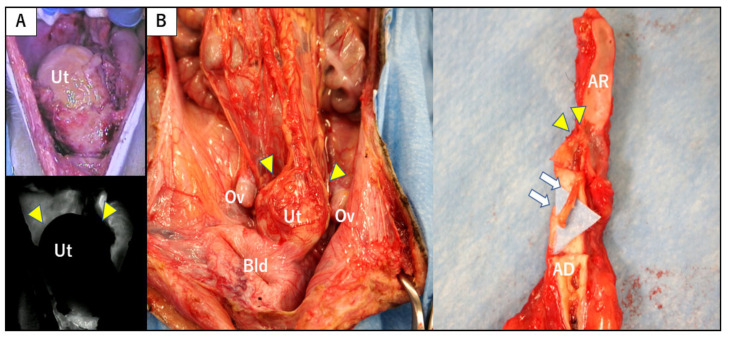
Macroscopic findings from autopsy of cynomolgus macaques with humoral rejection. (**A**) ICG fluorescent angiography of the transplanted uterus in autopsy of case 4. Enhancement of the grafted uterus (yellow triangles) was absent in the image (before (upper panel) and after (lower panel) injection of ICG). (**B**) Macroscopic findings of the abdomen in the autopsy of case 5, showing a whitish swollen transplanted uterus (yellow triangles) with severe adhesion to the surrounding tissues (left panel). The grafted aorta was along the long axis and intravascular thrombi (white arrow) were found in the anastomosis site of aortas (yellow triangles) to the grafted common iliac arteries (right panel). AD, aorta of the donor; AR, aorta of the recipient; Bld, bladder; OV, ovary; Ut, uterus.

Case 4, whose MHC was mismatched with that of the donor, was positive for DSA on POD 65 and notable enlargement of grafted uterus was seen in its transabdominal ultrasonography. A uterine biopsy on POD 67 showed fibrin thrombi throughout specimens without definitive evidence of tissue necrosis, indicating low-grade humoral rejection. Although steroid pulse therapy and ATG were administered, a biopsy showed extensive coagulation necrosis of uterine tissue on POD 83. Consequently, an autopsy was performed on POD 103. Macroscopic findings of the abdomen showed a whitish swollen transplanted uterus with severe adhesion to surrounding tissues. Subsequent ICG fluorescence angiography showed no uterine blood flow (Figure 6A). A hypertrophic myometrium was found in the resected uterus and an abscess was found in the uterine cavity. Histopathological findings revealed infarction of the uterus due to concentric severe stenosis of aortic graft and smaller arteries—consistent with findings of end stage of humoral rejection.

Case 5, whose MHC was mismatched with that of the donor, was positive for DSA on POD 33, and had an atrophic uterus, as revealed via transabdominal ultrasonography. Steroid pulse therapy was used even though uterine rejection was not shown. MMF was replaced with azathioprine on POD 145 due to recovery of periodic menstruation. However, on POD 206 transabdominal ultrasonography indicated that the grafted uterus had enlarged, whereupon a biopsy showed regional necrosis and fibrin thrombi. Subendothelial C4d staining was observed in blood vessels. As these findings suggested high-grade humoral rejection, treatment was administered via steroid pulse therapy and rituximab (10 mg/kg). However, findings of humoral rejection were observed in the subsequent biopsy and an autopsy was performed on POD 265. Laparotomy results showed a swollen white uterus that adhered to the omentum and to vascular sites surrounding the aorta and IVC (Figure 6B). An abscess was found in the uterine cavity and thrombi were detected in the grafted intravascular space from anastomosis site of aortas to the grafted common iliac arteries (Figure 6B). Extensive necrosis was observed throughout the uterus, along with severe arterial stenosis within graft tissues.

### 3.4. Post-Transplant Lymphoproliferative Disorder

**Figure 7:**
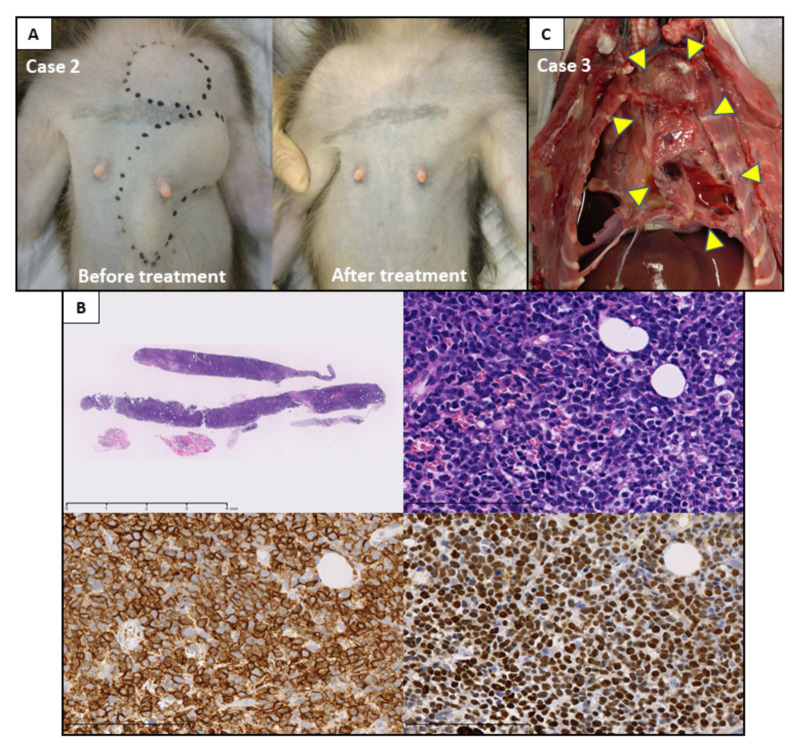
Development of PTLD with long-term graft survival after uterus transplantation in cynomolgus macaques. (**A**) Case 2 developed PTLD tumors in the anterior thorax and axilla on postoperative day (POD) 180 (left panel). The tumors disappeared completely one month following treatment due to repetitive administering of rituximab and cessation of MMF and TAC (right panel). (**B**) Biopsy specimen diagnosed as PTLD. Loupe image of needle biopsy specimens (H&E stain, bar = 4 mm); (upper left panel). Proliferation of atypical large lymphocytic cells are observed (H&E stain, bar = 100 μm); (upper right panel). Large atypical cells are positive for CD20; bar = 100 μm (lower left panel). Large atypical cells are positive for EBER in situ hybridization, bar = 100 μm (lower right panel). (**C**) Autopsy of case 3 showing tumors (yellow triangles) in the pleural cavity and mediastinum, diagnosed as PTLD.

Case 2 developed tumors in the anterior thorax and axilla on POD 180 (Figure 7A). A biopsy of the tumors showed diffuse infiltration by large lymphocytic cells, which were positive for CD20 and EBER-ISH, pointing to a diagnosis of PTLD, B-cell type (Figure 7B). Repetitive administration of rituximab (20 mg/kg; 4 times) and cessation of MMF and TAC aimed at eliminating tumors achieved a complete response without recurrence (Figure 7A). The autopsy of case 3, which suffered a sudden death on POD 170, incidentally showed tumors in the pleural cavity and mediastinum (Figure 7C). Histological examinations revealed EBER-negative PTLD, B-cell type.

### 3.5. Immunological Studies

#### 3.5.1. Changes in Peripheral Lymphocyte Counts

**Figure 8:**
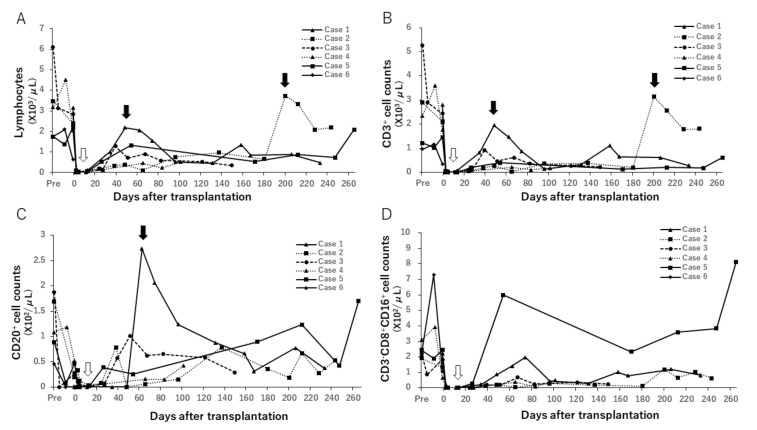
Changes in peripheral lymphocyte counts. Peripheral lymphocyte (**A**), CD3+ (**B**), CD20+ (**C**) and NK cell (**D**) counts decreased after induction treatment with antithymocyte globulin (ATG) and rituximab (white arrow). CD3+ and CD20+ counts gradually increased following induction treatment, and then increased remarkably with rejection in some cases (black arrow). In case 2, lymphocyte and CD3+ cell counts elevated on POD 200 with low-grade cellular rejection caused by cessation of TAC and MMF due to treatment for PTLD, while CD20+ was suppressed due to administering of rituximab for PTLD.

Treatment via induction of ATG and administration of rituximab decreased peripheral lymphocyte, CD3+, CD20+ and NK (CD3-CD8+CD16+) cell counts immediately following surgery (Figure 8). CD3+ and CD20+ counts showed a gradual increase after approximately 1 month, evident increase in correspondence to rejection, and decreased following treatment. Regulatory T cells (CD4+FOXP3+) were not associated with rejection and outcomes (data not shown).

#### 3.5.2. DSA

**Figure 9:**
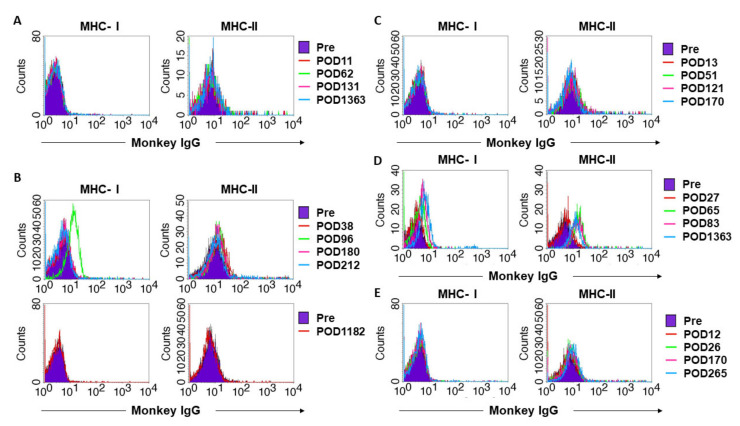
Antibody responses against donor cells in case 1 (**A**), case 2 (**B**), case 3 (**C**), case 4 (**D**), and case 5 (**E**). FITC-labeled anti-monkey IgG antibodies were used as secondary antibodies. After gating at CD3-positive lymphocytes and CD20-positive lymphocytes, we made FITC-histograms to analyze IgG specific for each lymphocyte. Histograms of MHC-I (left) and MHC-II (right) exhibiting IgG antibodies specific for donor cells gated by CD3+ cells and CD20+ cells, respectively. In case 2, DSA specific for MHC class II was detected on POD 38, in addition to which DSA specific for MHC class I was detected on POD 48 (data not shown). Higher levels of DSA specific for MHC class I was detected on POD 96. Thereafter, the titer of MHC-class I-specific DSA gradually decreased and vanished on POD 180 when PTLD occurred, while low levels of MHC class II-specific DSA remained (**B**; upper panel). After PTLD was treated, MHC class I and II-specific DSA were not detected until delivery during the period from POD 212 to POD 1182 (**B**; lower panel). In case 4, in which a uterus with full-mismatched MHCs was transplanted, DSA was detected on POD 65 and similar levels of DSA was also detected on POD 83 and 101 (**D**). Low levels of MHC class I and II-specific DSA were detected in case 5, in which a uterus with mismatched MHC, except for one locus of MHC class IB, was transplanted (**E**). DSA was not detected in cases 1 and 3, in which multiple loci of MHC were matched between donor and recipient. (**A**,**C**).

DSA was detected on POD 38, 65, and 26 in case 2, 4, and 5, respectively. MHC class I and II-specific DSAs in case 2 were eliminated following the treatment of PTLD. DSA was not detected in cases 1 and 3, in which multiple loci of the MHCs were matched between donor and recipient (Figure 9).

#### 3.5.3. MLR

**Figure 10:**
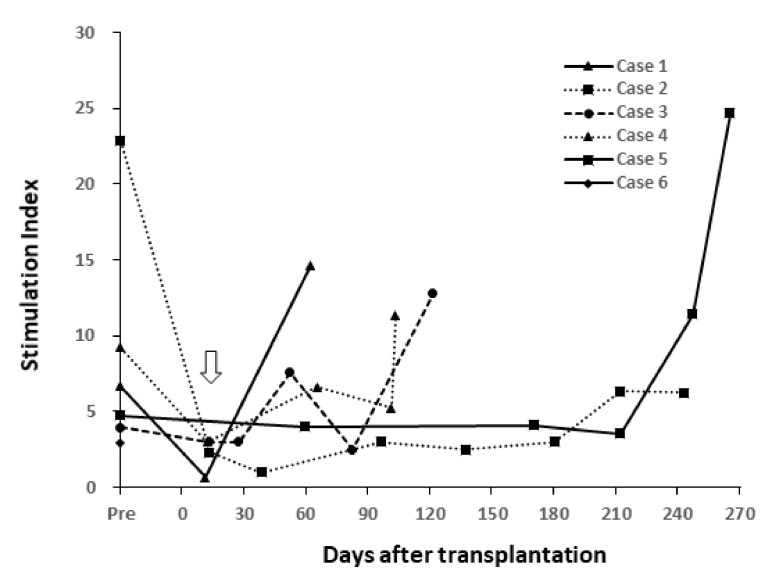
Mixed Lymphocyte Reaction (MLR) stimulation indexes in pre- and post-transplantation. The MLR stimulation index decreased following induction treatment (white arrow). This index increased in conjunction with rejection in cases 1, 4, and 5. The index on POD 1363 in case 1 and on POD 1184 (on delivery) in case 2 were 2.10 and 1.33, indicating that index levels were low (data not shown).

The MLR stimulation index against donor and third-party antigens was determined following surgery (Figure 10). This index decreased after induction treatment in all cases and showed a tendency to increase when rejection occurred.

### 3.6. Pregnancy and Delivery

**Figure 11:**
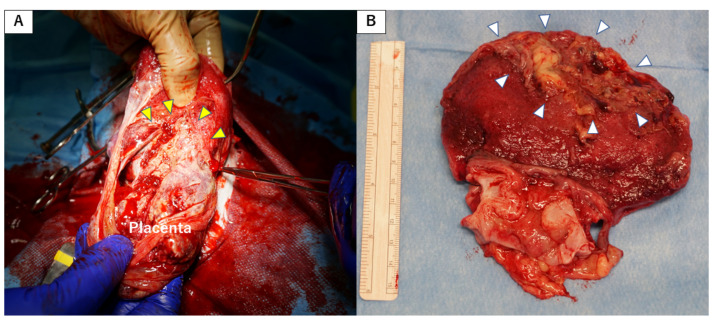
First pregnancy and delivery after allogeneic uterus transplantation in a cynomolgus macaque. (**A**) Fetal heartbeat as per Doppler ultrasonography. Fetal viability was confirmed by transabdominal ultrasonography in case 2, with a crown-rump length of 11.7 mm and fetal cardiac activity. (**B**) Intraoperative findings of the cesarean section. Transverse incision of the lower part of uterus was performed.

**Figure 12:**
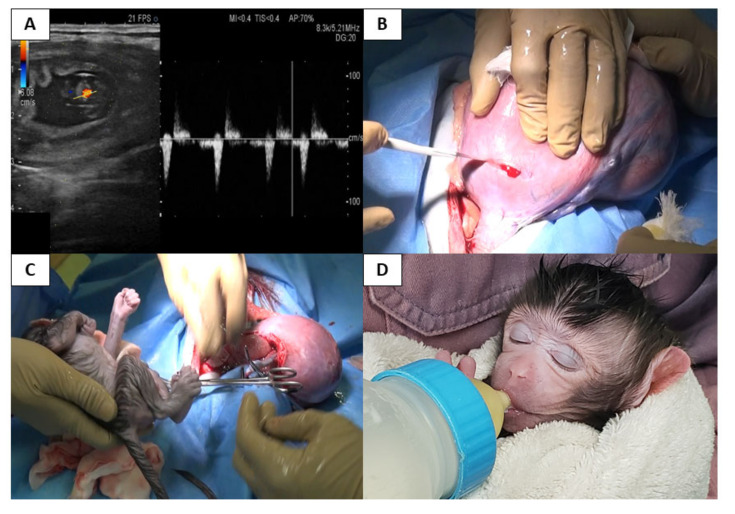
Placenta accreta in transplanted uterus. (**A**) Placenta accreta was observed intraoperatively during cesarean section. Placenta was firmly adhered to the fundus of transplanted uterus (yellow triangles). (**B**) Resected transplanted uterus. Rests of partial placenta was observed in the fundus of the transplanted uterus (white triangles). (**C**) Delivery of the offspring from the transplanted uterus. (**D**) First newborn offspring after allogeneic uterus transplantation in a cynomolgus macaque. The body weight of the offspring was 358.4 g at delivery, indicating satisfactory growth during the pregnancy.

In all cases menstruation resumed within 4 months after surgery, but only temporarily in case 4. Following confirmation of three cycles of periodic menstruation and no history of evident rejection via histopathology, replacement MMF with azathioprine followed by artificial insemination was performed in order to induce pregnancy in cases 1 to 3; however, pregnancy was not achieved. Therefore, ET was conducted from POD 572 and 356 onwards in case 1 and 2, respectively but not in case 3 due to sudden death. Despite 10 attempts at ET, pregnancy was not achieved in case 1. In case 2, the third ET performed on POD 417, which confirmed the first pregnancy when a fetus with cardiac activity was confirmed on POD 446 (Figure 11). However, a missed abortion occurred as indicated by the absence of a fetal heartbeat after one month. Laparotomy was conducted to remove intrauterine tissue. Thereafter, second pregnancy showing a fetal heartbeat was achieved on POD 734 via the initial ET following abortion. However, this heartbeat became undetectable after one week and removal resurgery was conducted two months after diagnosing abortion. Then, a third pregnancy was achieved via the first ET following the second abortion on POD 1063, in which a fetus with a heartbeat was confirmed using ultrasonography. The fetus grew appropriately during the pregnancy period and eventually a cesarean section was performed at full-term 152 days following fertilization, and a delivery following uterus transplantation in a cynomolgus macaque was successfully accomplished for the first time (Figure 11B,C; Video S1). Uterus contraction following delivery was good, and a placenta accreta was observed (Figure 12). The body weight of the offspring was 358.4 g with no abnormalities observed (Figure 12D). There were no episodes of rejection during the period starting from the attempt at pregnancy to delivery; biopsy was not conducted during pregnancy to avoid a potential abortion due to biopsy procedures.

## 4. Discussion

To the best of our knowledge, this is the first ever successful delivery following allogeneic uterus transplantation in cynomolgus macaque (nonhuman primates). Notably, this delivery was accomplished after recovery from PTLD and DSA coincident with graft rejection.

Due to anatomic and physiologic similarities between the reproductive organs and immune systems of nonhuman primates and humans, UTx studies on nonhuman primates provide important preclinical data that may help to resolve issues associated with UTx in humans. However, some difficulties involving postoperative control are encountered due to certain limitations, including size; administration of drugs; ultrasonography, biopsies, and blood tests; fertilization; and embryo transfers in these animals [31,32]. As there are no previous reports on the use of rituximab in human UTx, the current study enhanced the immunosuppressive regimen by adding rituximab to the previous protocol [14]. There are no reports indicating previous use of rituximab in human UTx. Moreover, donor and recipient animals with haploidentical MHCs were used to reduce graft rejection.

PTLD is an abnormal lymphoid proliferation occurring in depressed T-cell function due to prolonged immunosuppression. Activated functional T cells—which play an important role in regulation of EBV-infected cells—is impaired in transplant recipients and may lead to EBV-associated PTLD [33,34]. The incidence of PTLD appears to be tissue specific, with rates as high as 20% in recipients of small intestine transplants, 10% in lung transplants, and 1–6% for heart, liver, and kidney transplants [35]. The different levels of PTLD that have been reported in cynomolgus macaques are as follows: 9 of 160 (5.6%) in a renal transplantation model [36]; 9 of 28 (32%) in neuronal xenotransplantation model [37]; and 5 of 10 (50%) in a composite facia allografts model [38]. Herein, a high frequency of PTLD was observed in immunosuppressed macaques following UTx; 2 of 5 (40%, case 6 is excluded) in the present study and 1 of 4 (25%) in the previous study. However, there are no reports showing that PTLD in cynomolgus macaques responds completely to treatment. Rituximab is the standard treatment for PTLD patients, who do not respond to reduced immunosuppression, with complete remission rates ranging from 20 to 55% [39]. We administered rituximab repetitively and reduced immunosuppression to treat PTLD in case 2, resulting in a complete response. This is the first report of successful recovery from PTLD following UTx in animal models.

Furthermore, various features of uterine rejection, which developed despite the strengthening of immunosuppression, were observed in this study. Notably, humoral rejections coincident with the DSA previously reported [14] were also detected in some animals by this study. Although mild, steroid-responsive T-cellular rejection is the form mostly reported in human UTx, humoral rejection coincident with DSA has also been recently reported by a team at the Cleveland Clinic [40]. Based on our experience of UTx research using cynomolgus macaques that have been conducted so far, we believe that rejection of humoral components is more likely to develop in cynomolgus macaques, whose uteri may exhibit high levels of antigenicity, compared with those of humans. Herein, three of five (60%, excluding case 6) produced DSA and two of those three developed humoral rejections leading to uterine necrosis. In our previous study, three of four (75%) produced DSA and all three experienced graft failure [14]. Surprisingly, DSA detected concurrently with cellar rejection in case 2 disappeared following rituximab treatment for PTLD, resulting in a successful delivery. Based on our experience of various features that accompany uterine rejection in cynomolgus macaques, including cellular and humoral component rejection, we established new pathological rejection criteria, by revising previous criteria proposed by Mölne et al. [27] and subsequently evaluated uterine rejection. As humoral rejection is aggressive and presents a serious threat to graft viability, minute attention to monitoring and detection of its features is required to enhance human UTx procedures in the future.

Notably, the current study used MHC-defined macaques only in an exploratory manner to investigate the reduction of uterine rejection and the association between MHC matching and rejection. Importantly, severe uterine rejection did not occur in animals carrying haploidentical MHCs compared with those carrying mismatched MHCs, wherein one animal (Case 2) in the former category achieved successful delivery, while the animals in the latter category experienced multiple cellular and humoral rejections. Preoperative MLR stimulation index was high (22.9) for Case 2; however, we could not figure out the reason for this. Prior to UTx, it might have been exposed to an antigen similar to the donor’s MHC. Although verification via a few animals may be considered as a limitation, MHC (HLA) compatibility likely influences graft survival in UTx similar to that in other organs although this has yet to be demonstrated in humans and there are apparently few problems associated with uterine rejection even in the case of mismatched HLA between the donor and recipient in previous reports of human UTx. Therefore, findings of the current study indicate that cynomolgus macaque MHC-defined UTx models may help to elucidate the nature of immune response following allogeneic UTx or other allogenic transplantations [22,23,24]. However, the difference in outcomes of the UTx model of cynomolgus macaques compared to humans seems to be related to the difference in immune response rather than to the surgical technique. Uterine rejection is more frequent in cynomolgus macaques than has been previously reported for humans. This is a limitation that human UTx could not be completely mimicked in this study.

Interestingly, as reported in one human UTx case [40], placenta accreta was observed in the transplanted uterus upon delivery. No increased incidence of placenta accreta has been reported in pregnancy following organ transplantation. Moreover, development of abruptio placentae upon delivery following autologous UTx has also been observed in a macaque [18]. It remains unknown whether these abnormal placentas are associated with grafted uteri or immunosuppression, although assisted reproductive technology is an acknowledged risk factor [41]. Thus, UTx related experimentation may lead to further scientific studies pertaining to maternal-fetal-placental relationships, immunology, and reproductive biology.

## 5. Conclusions

The current study achieved the first successful delivery following allogeneic UTx, during which PTLD and DSA coincident with graft rejection occurred—but it was successfully managed. At present, such a delivery is possible only with UTx between a pair carrying haploidentical MHCs, due to high antigenicity seen in macaque uteri. UTx is a field in development and as with other transplant fields, large animals and nonhuman primates are required to test hypotheses before systematic study in humans. Importantly, experimental data obtained from nonhuman primate studies will produce valuable new scientific knowledge that may enable the resolution of yet unsolved clinical and technical issues associated with UTx. Therefore, verification via basic animal models should be performed in conjunction with clinical trials to gain further insight into issues relating to safety and efficiency that could be applied to human UTx.

## Figures and Tables

**Table 1:** Pretransplant characteristics of the study subjects.

Case	Donor					Recipient						MHC Compatibility between Donor and Recipient
	Blood Type	Age (Years)	Weight (kg)	Parity	EBV IgG Antibody	Blood Type	Age (Years)	Weight (kg)	Parity	DSA	EBV IgG Antibody	
1	A	6	3.22	no	+	A	10	3.64	no	−	+	mismatched*
2	B	9	3.39	no	+	B	9	3.7	no	−	+	halpoidentical
3	B	5	4.07	no	+	B	7	3.45	no	−	+	halpoidentical
4	B	12	3.76	one	+	B	13	3.34	no	−	+	mismatched
5	AB	5	2.87	no	+	AB	6	3.56	no	−	+	mismatched
6	B	6	2.67	no	+	B	6	3.09	no	−	+	mismatched

* Mafa-DQB1 and -DPB1 are haploidentical in case 1. EBV, Epstein–Barr virus.

**Table 2:** Genotyping data of cynomolgus macaque MHC (Mafa) genes in case 2.

Donor/Recipient	Donor		Recipient	
Origin	Filipino		Filipino	
Mafa-A	A1*089:03	A1*094:01	A1*089:03	A1*094:01
	A2*05:50	A2*05:04	A2*05:50	A2*05:04
	A3*13:03	-	A3*13:03	-
Mafa-B	B*033:02	B*045:05	B*033:02	B*041:01
	B*095:01	B*098:08	B*095:01	B*048:03
		B*099:01		B*060:02
		B*108:01		B*089:01
Mafa-DRB	DRB1*03:21	DRB1*03:07	DRB1*03:21	DRB*W53:01
	DRB1*10:07	DRB1*10:06	DRB1*10:07	DRB*W54:01
Mafa-DQB1	DQB1*06:08	DQB1*06:25	DQB1*06:08	DQB1*06:35
Mafa-DPB1	DPB1*15:04	DPB1*19:03	DPB1*15:04	DPB1*03:04

* Underlined alleles indicate alleles that were identical between donor and recipient pairs.

**Table 3:** Immunosuppressive protocol used in this study.

	Rituximab	Antithymocyte Globulin	Tacrolimus	Mycophenolate Mofetil	Methylprednisolone	Azathioprine
Three weeks before surgery	2 mg/kg					
⋮						
day-5			↓	↓		
⋮			↓	↓		
day 0 ^a^	2 mg/kg	20 mg/kg	↓	↓	10 mg/kg	
day 1			↓	↓	10 mg/kg	
day 2		20 mg/kg	↓	↓	5 mg/kg	
⋮			administered with target trough levels of 15 to 20 mg/mL during first month and tapered thereafter as required	administered orally twice daily and adjusted thereafter to exceed trough levels of at least 1 µg/mL	gradually tapered and continuously administered at daily doses of 0.2–0.4 mg/kg, until the end of observation	Mycophenolate mofetil was replaced with azathioprine (1–2 mg/kg) before impregnation was attempted

^a^ day 0; the day of surgery.

**Table 4:** Histologic criteria for cellular and humoral rejection.

A. Criteria for Cellular Rejection		
Histologic Decision	Stromal Features	Epithelial Features *
Not rejection	None or focal perivascular inflammation. <S0>	No significant findings. <E0>
Borderline change	Diffuse perivascular inflammation without endotheliitis. <S1>	Focal or diffuse vacuolar alteration of the basal cell layer with or without small foci indicative of junctional inflammation. <E1>
Cellular rejection, low-grade	Inflammation with focal endotheliitis. <S2>	Dyskeratotic squamous cells in the epidermis with basal vacuolar alteration. <E2>
Cellular rejection, high-grade	Inflammation with diffuse endotheliitis. <S3>	Subepidermal cleft, or complete separation of epithelium from the stroma. <E3>
**B. Criteria for Humoral Rejection**		
**Histologic Decision**	**Arterial Features**	
Not rejection	None or focal perivascular inflammation. <A0>	
Humoral rejection, low-grade	A few fibrin thrombi and/or fibrinoid change of the small vessels without tissue necrosis. <A1>	
Humoral rejection, high-grade	Marked fibrin thrombi with tissue necrosis. <A2>	

Note: Adaptable only for specimens without humoral rejection. Focal, 0%< and <50%; diffuse, > = 50%. *Only squamous epithelium should be evaluated.

**Table 5:** Outcomes of clinical course in recipients.

Case	Survival (Days)	Recovery of Menstruation	Method of Impregnation	Pregnancy/Delivery	Maximum Cellular Rejection Episode	Humoral Rejection	DSA Production	MLR Stimulation Indexes (Pretransplant; Maximum)	Cause of Sacrifice
1	1400 < on going	Periodic	AI ICSI-ET	−	low-grade cellular rejection	−	−	6.6; 14.7	N/A
2	1184	Periodic	AI ICSI-ET	2 abortions/ 1 livebirth	low-grade cellular rejection	−	+	22.9; 22.9	Upon cesarean section
3	170	Periodic	AI	−	Borderline	−	−	3.9; 12.8	Sudden death induced by PTLD and hyperpotassemia
4	103	Temporary	−	−	−	high-grade humoral rejection	+	9.2; 11.3	Humoral rejection
5	265	Periodic	−	−	−	high-grade humoral rejection	+	4.7; 24.7	Humoral rejection
6	0	N/A	N/A	N/A	N/A	N/A	N/A	2.9; N/A	Infusion associated reaction

AI, artificial insemination; ICSI-ET, intracytoplasmic sperm injection and embryo transfer; N/A, not applicable; PTLD, post-transplant lymphoproliferative disorder.

## Supplementary Materials

The following are available online at https://www.mdpi.com/2077-0383/9/11/3694/s1, Table S1: Genotyping data of cynomolgus macaque MHC (Mafa) genes in six uterus transplantation cases, Table S2: Antibodies for flow cytometry, Table S3: Surgical parameters for allogeneic uterus transplantation in cynomolgus macaques, Video S1: The first ever delivery following allogeneic uterus transplantation in a cynomolgus macaque.
