# Supplementary material for: First Successful Delivery after Uterus Transplantation in MHC-Defined Cynomolgus Macaques
Source: J Clin Med. 2020 Nov 17;9(11):3694. doi: 10.3390/jcm9113694 (PMC7698480; doi:10.3390/jcm9113694)
Supplement: Supplementary file 1 [file jcm-09-03694-s001.zip › Supplementary Materials/Table S1.docx]

*Supplementary Table 1. Genotyping data of cynomolgus macaque MHC (Mafa) genes in six uterus transplantation cases*

|  |  |  |  |  |  |  |  |  |  |  |  |  |  |  |  |  |  |
| --- | --- | --- | --- | --- | --- | --- | --- | --- | --- | --- | --- | --- | --- | --- | --- | --- | --- |
| Case No. | Case 1 | |  | Case 2 | |  | Case 3 | |  | Case 4 | |  | Case 5 | |  | Case 6 | |
| Donor/Recipient | Donor | Recipient |  | Donor | Recipient |  | Donor | Recipient |  | Donor | Recipient |  | Donor | Recipient |  | Donor | Recipient |
| Origin | Chinese | Chinese |  | Filipino | Filipino |  | Filipino | Filipino |  | Chinease | Filipino |  | Vietnamese | Filipino |  | Cambodian | Cambodian |
| Mafa-A | A1*003:03 | A1*087:02 |  | A1*089:03 | A1*089:03 |  | A1*004:01 | A1*089:03 |  | A1*079:02 | A1*052:02 |  | A1*061:01 | A1*008:02 |  | A1*022:06/10 | A1*065:04 |
|  | A1*065:03 | A1*097:01 |  | A1*094:01 | A1*094:01 |  | A1*089:03 | A2*05:50 |  | A1*097:01 | A1*098:02 |  | A1*065:04:01 | A1*086:02 |  | A1*022:11 | A1*070:01 |
|  | A2*05:06/43 | A2*05:06/43 |  | A2*05:04 | A2*05:04 |  | A2*05:50 | A3*13:03 |  | A2*05:25:01 | A2*24:06 |  | A4*14:03/04 | A1*094:01 |  | A1*066:06 | A3*13:20 |
|  | A2*05:40 | A3*13:15 |  | A2*05:50 | A3*13:03 |  | A3*13:03 |  |  | A2*05:44 | A4*01:04 |  |  | A2*05:04 |  | A4*14:03/04/13 |  |
|  | A3*13:07 | A3*13:19 |  | A3*13:03 |  |  |  |  |  | A3*13:15 |  |  |  |  |  |  |  |
|  | A4*14:03/04_new |  |  |  |  |  |  |  |  | A4*14:03/04 |  |  |  |  |  |  |  |
| Mafa-B | B*013:04_new | B*007:01 |  | B*033:02 | B*033:02 |  | B*033:02 | B*033:02 |  | B*013:03 | B*017:01 |  | B*030:01/03/07 | B*030:14 |  | B*007:01/07/09/11 | B*019:03/05 |
|  | B*013:06 | B*013:03 |  | B*045:05 | B*041:01 |  | B*079:02 | B*050:08 |  | B*030:02 | B*046:01 |  | B*044:01:01 | B*046:01 |  | B*038:01 | B*023:03 |
|  | B*014:01 | B*051:05 |  | B*098:08 | B*048:03 |  | B*085:01 | B*056:02 |  | B*043:01 | B*050:08 |  | B*060:03_new | B*050:08 |  | B*051:05/08 | B*030:05 |
|  | B*034:03 | B*056:01 |  | B*099:01 | B*060:02 |  | B*095:01 | B*060:03 |  | B*057:03 | B*056:02 |  | B*079:02 | B*057:04 |  | B*060:04/13/19 | B*035:01 |
|  | B*050:05 | B*060:03_new |  | B*095:01 | B*089:01 |  | B*158:01 | B*089:01 |  | B*073:01 | B*057:04 |  | B*105:01 | B*060:02 |  | B*101:02 | B*079:02 |
|  | B*060:10 | B*070:01/02_new |  | B*108:01 | B*095:01 |  | B*159:01 | B*157:01 |  | B*092:01 | B*060:02 |  | B*149:02 | B*065:03 |  |  | B*079:06 |
|  | B*068:03 | B*088:02_new |  |  |  |  |  | B*095:01 |  |  | B*060:03 |  | B*156:01 | B*072:01 |  |  | B*085:01 |
|  | B*068:03_new | B*137:06/07/08 |  |  |  |  |  |  |  |  | B*072:01 |  |  | B*079:02 |  |  |  |
|  | B*091:01_new |  |  |  |  |  |  |  |  |  | B*089:01 |  |  | B*104:03 |  |  |  |
|  | B*115:02 |  |  |  |  |  |  |  |  |  | B*104:03 |  |  | B*114:02 |  |  |  |
|  |  |  |  |  |  |  |  |  |  |  | B*114:02 |  |  | B*144:03N |  |  |  |
|  |  |  |  |  |  |  |  |  |  |  | B*116:01 |  |  | B*161:04 |  |  |  |
|  |  |  |  |  |  |  |  |  |  |  | B*144:03N |  |  |  |  |  |  |
|  |  |  |  |  |  |  |  |  |  |  | B*157:01 |  |  |  |  |  |  |
| Mafa-DRB | DRB*W20:03 | DRB*W001:13/15 |  | DRB1*03:07 | DRB*W53:01 |  | DRB*W53:01 | DRB1*03:21 |  | DRB*W3:09 | DRB*W33:02 |  | DRB*W4:04 | DRB*W1:08 |  | DRB*W026:01 | DRB*W001:01 |
|  | DRB*W26:01 | DRB*W3:04 |  | DRB1*03:21 | DRB*W54:01 |  | DRB*W54:01 | DRB1*10:07 |  | DRB*W21:01 | DRB1*04:04 |  | DRB*W26:01 | DRB*W3:01 |  | DRB*W027:08 | DRB*W002:06 |
|  | DRB1*03:01 | DRB*W3:05 |  | DRB1*10:06 | DRB1*03:21 |  | DRB1*03:21 |  |  | DRB*W27:01 | DRB5*03:02 |  | DRB*W27:04 | DRB*W36:01 |  | DRB*W066:02 | DRB*W002:08 |
|  | DRB1*03:12/36 | DRB*W66:02 |  | DRB1*10:07 | DRB1*10:07 |  | DRB1*10:07 |  |  | DRB*W66:03 |  |  | DRB1*03:12 | DRB1*03:21 |  | DRB1*03:06 | DRB1*03:07 |
|  |  | DRB1*03:03/30 |  |  |  |  |  |  |  |  |  |  | DRB3*04:03 | DRB1*10:07 |  | DRB1*03:12/36 | DRB1*04:15 |
| Mafa-DQB1 | DQB1*06:14 | DQB1*15:03 |  | DQB1*06:08 | DQB1*06:08 |  | DQB1*06:08 | DQB1*06:08 |  | DQB1*18:04 | DQB1*18:27 |  | DQB1*06:14 | DQB1*06:08 |  | DQB1*06:14 | DQB1*06:13/44/45 |
|  | DQB1*18:17 | DQB1*18:17 |  | DQB1*06:25 | DQB1*06:35 |  | DQB1*06:35 |  |  | DQB1*18:08 |  |  | DQB1*18:04 | DQB1*18:07 |  | DQB1*18:04 | DQB1*17:03 |
| Mafa-DPB1 | DPB1*01:09 | DPB1*06:07 |  | DPB1*15:04 | DPB1*03:04 |  | DPB1*03:04 | DPB1*15:04 |  | DPB1*01:04 | DPB1*08:04 |  | DPB1*15:01 | DPB1*10:01 |  | DPB1*15:01/13 | DPB1*01:02/11/14 |
|  | DPB1*15:01 | DPB1*15:01 |  | DPB1*19:03 | DPB1*15:04 |  | DPB1*15:04 |  |  | DPB1*18:01 |  |  | DPB1*19:06 | DPB1*15:04 |  | DPB1*18:01/03 | DPB1*18:02 |

*Underlined alleles indicate alleles that are identical between donor and recipient pairs.
