# Supplementary material for: First Successful Delivery after Uterus Transplantation in MHC-Defined Cynomolgus Macaques
Source: J Clin Med. 2020 Nov 17;9(11):3694. doi: 10.3390/jcm9113694 (PMC7698480; doi:10.3390/jcm9113694)
Supplement: Supplementary file 1 [file jcm-09-03694-s001.zip › Supplementary Materials/Table S2.docx]

*Supplementary Table 2. Antibodies for flow cytometry*

|  |  |  |  |  |  |  |
| --- | --- | --- | --- | --- | --- | --- |
| **Antibody** | **Dye** | **Clone** | **Vendor** | **Isotype control** | **Clone** | **Vendor** |
| **CD3** | PE-Cy7 | SP34-2 | BD | Mouse IgG1 κ | MOPC-21 | BD |
| **CD4** | FITC | OKT4 | BioLegend | Mouse IgG2b κ | MPC-11 | BioLegend |
| **CD4** | APC | OKT4 | BioLegend | Mouse IgG2b κ | MG2b-57 | BioLegend |
| **CD8** | BV510 | RPA-T8 | BioLegend | Mouse IgG1 κ | MOPC-21 | BioLegend |
| **CD16** | V450 | 3G8 | BD | Mouse IgG1 κ | X40 | BD |
| **CD20** | PE | 2H7 | BioLegend | Mouse IgG2b κ | MPC-11 | BioLegend |
| **FOXP3** | PE | 236A/E7 | eBioscience | Mouse IgG1 κ | MOPC-21 | BD |

*Abbreviations: PE-Cy7: phycoerythrin-cyanin 7, FITC: fluorescein isothiocyanate, APC: Allophycocyanin, BV510: Brilliant Viole 510, V450: Violet 450, PE: phycoerythrin, BV421: Brilliant Violet 421, BD: Becton, Dickinson and Company including BD bioscience and BD pharmingen*
