# Supplementary material for: First Successful Delivery after Uterus Transplantation in MHC-Defined Cynomolgus Macaques
Source: J Clin Med. 2020 Nov 17;9(11):3694. doi: 10.3390/jcm9113694 (PMC7698480; doi:10.3390/jcm9113694)
Supplement: Supplementary file 1 [file jcm-09-03694-s001.zip › Supplementary Materials/Table S3.docx]

*Supplementary Table 3. Surgical parameters for allogeneic uterus transplantation in cynomolgus macaques.*

|  |  |  |  |  |  |  |
| --- | --- | --- | --- | --- | --- | --- |
| Case | Duration of  procurement surgery | Duration of  recipient surgery | Total  ischemic time | Warm  ischemic time* | Cold  ischemic time^†^ | Time required  for vascular anastomosis |
| 1 | 7h 27min | 5h 46min | 1h 59min | 1h 33min | 26min | 1h 15min |
| 2 | 7h 38min | 5h 51min | 2h 11min | 1h 28min | 43min | 55min |
| 3 | 8h 3min | 5h 53min | 1h 56min | 1h 19min | 37min | 1h 2min |
| 4 | 7h 48min | 6h 39min | 2h 4min | 1h 33min | 31min | 1h 2min |
| 5 | 7h 45min | 5h 40min | 2h 2min | 1h 32min | 30min | 1h 5min |
| 6 | 8h 25min | 5h 2min | 1h 43min | 1h 23min | 20min | 58min |
| Average | 7h 51min | 5h 38min | 1h 59min | 1h 28min | 31min | 1h 2min |

* Warm ischemia in the recipient was defined as the time from cross-clamping at organ recovery to commencement of cold flushing and from removal of the uterine graft out of the cold preservation solution to reperfusion in the recipient.

† Cold ischemia was defined as the time from commencement of cold perfusion in the donor to the removal of the graft from the cold preservation solution.
